# Taxonomic Composition, Trade Networks, and Consumer Demand in China’s Illegal Bird Trade: Evidence from Court Verdicts (2014–2021)

**DOI:** 10.3390/biology15212015

**Published:** 2026-11-05

**Authors:** Yuna Zhang, Tianjian Song, Ye Chen, Yuxin Huang, Zexu Luo, Yuxi Zhuang, Jiang Chang

**Affiliations:** 1 State Key Laboratory of Environmental Criteria and Risk Assessment, Chinese Research Academy of Environmental Sciences, Beijing 100012, China; zyn1106877099@163.com (Y.Z.); 19505568068@163.com (Y.C.); hyx573542801@126.com (Y.H.); zhuangyx2025@lzu.edu.cn (Y.Z.); 2 College of Ecology, Lanzhou University, Lanzhou 730000, China; 3 School of Environment and Energy, Peking University Shenzhen Graduate School, Shenzhen 518055, China; tjian_song@163.com; 4 College of Life Sciences, Anhui Normal University, Wuhu 241000, China; 5 Institute of Zoology, Chinese Academy of Sciences, 1 Beichen West Road, Chaoyang, Beijing 100101, China; 20162680010@m.scnu.edu.cn

**Keywords:** illegal wildlife trade, bird, network analysis, consumer demand

## Abstract

Illegal wildlife trade (IWT) is a major driver of biodiversity loss, and birds are among the most heavily trafficked taxa. Although China has one of the world’s largest wildlife markets, the spatial, temporal, and taxonomic structure of its domestic illegal bird trade remains poorly characterized. We retrieved and analyzed 1427 first-instance criminal verdicts issued between 2014 and 2021 from China Judgments Online, using descriptive and trade-network analyses to identify the key traded species, temporal patterns and major trading regions. The trade involved 19 orders, 48 families and 266 species, of which 37 were listed as threatened (CR, EN and VU) on the Red List of China’s Vertebrates and 24 were Class I nationally protected. Psittaciformes appeared in the greatest number of verdicts, and the recorded purposes indicate that they were traded mainly to meet experiential demand. Annual verdict counts rose steadily from 2014 to 2019 and then declined sharply, reaching 40 in 2021. Illegal bird trade was documented in 30 provincial-level regions and was concentrated in southwestern and southeastern China. Weighted by the number of verdicts, Guangdong was the leading province and Guangzhou the leading Chinese prefecture. Of the 1001 verdicts with a geolocatable origin and destination, 90.81% involved transactions over distances of less than 500 km. These findings improve understanding of China’s illegal bird trade and can inform law enforcement and public-awareness campaigns.

## Simple Summary

Illegal wildlife trade threatens biodiversity worldwide, and birds are among the most affected taxa. We analyzed 1427 court verdicts from 2014 to 2021 to identify which species were traded most frequently, how trade patterns changed over time, where the major trading hubs were located, and why people purchased these birds. Trade activity rose until 2019 and then declined sharply. Traded taxa spanned 19 orders, 48 families, and 266 species. Psittaciformes appeared in the greatest number of verdicts, mainly reflecting consumer demand for pets and personal enjoyment, whereas Galliformes were traded mainly for financial gain. Guangdong in southern China served as the main hub for both supply and demand, followed by Yunnan in the southwest. Most transactions involved short-distance movement between neighboring areas.

## 1. Introduction

Illegal wildlife trade (IWT) is one of the most lucrative environmental crimes, second only to arms and drug trafficking [1]. It harms the environment, society, and governance [2]. IWT has emerged as a major threat to global biodiversity, causing population declines and species extinctions [3,4]. Wildlife crime also increases the risk that alien species become established in new regions and is a primary pathway of biological invasions [5,6]. Furthermore, emerging infectious diseases in human populations originate in animals, mostly wildlife [7,8].

There are over 10,000 bird species globally, of which nearly half are directly exploited by humans [9]. China is exceptionally rich in bird species and has a long history of keeping them as pets. While captive breeding of birds can theoretically serve as an alternative to wildlife, research indicates that consumers prefer wild-caught birds because captive-bred birds are in short supply, cost more, and are considered poorer singers. Consequently, wild populations still face significant pressure from illegal trade [10]. Current research on bird trade consists mainly of market surveys in selected provinces or cities [11,12,13,14,15], and research on illegal bird trade networks has focused mainly on the global scale [16].

In the Neotropics, where illegal parrot trade has been studied most intensively, surveys of city markets, rural households and seizures indicate that poaching is driven mainly by local demand for pet birds rather than by international trade [17]. This demand can be substantial: in Bolivia alone, an estimated 300,000–500,000 parrots are poached each year, 20–70 times the number traded in the country’s main market [18], and household-poached parrots were recorded in 96% of the rural localities surveyed in Peru and southern Ecuador [19].

Wildlife smuggling in China is widespread in coastal and border regions, with Guangdong, Yunnan and Fujian acting as major transport hubs or border crime corridors, whereas smuggling rates decline markedly in the inland central and western provinces. The economically developed southeastern coast has facilitated the further expansion of smuggling networks [20]. The southeastern coast, together with eastern and northern China, forms high-density core areas for wildlife trafficking through transport terminals [21]. Illegal wildlife trade is predominantly intra-provincial, and in the Yunnan-Guangxi region it flows mainly toward southeastern coastal markets in Guangdong and Fujian [22]. Illegal trade in pangolins in China is likewise frequently recorded in Guangdong, Yunnan and Guangxi [23].

As societies have developed, patterns of wildlife consumption have changed substantially. Economic growth and the expansion of high-income populations have rapidly increased the consumption of high-value wildlife [24]. In addition, the cultural and social backgrounds of consumers shape their preferences for birds, thereby raising or lowering demand for particular species [25,26]. Research has addressed key knowledge gaps in consumer motivation and has produced a comprehensive framework that identifies five categories of motivation for wildlife trade [27]: experiential demand (the desire to seek pleasure, provide novelty, or satisfy curiosity), social demand (the desire to enhance social status or gain social recognition), functional demand (the need to fulfill an everyday purpose or function), financial demand (the desire for financial gain or immediate profit) and spiritual demand (the desire to meet spiritual needs or to obtain protection, luck or good fortune in business and life, sometimes interlinked with cultural practices).

On this basis, we test two hypotheses. (H1) Psittaciformes are the most frequently traded group in China’s illegal bird trade, and their trade is driven mainly by experiential demand. (H2) China’s illegal bird trade is concentrated in coastal or border provinces such as Guangdong and Yunnan, and is dominated by short-distance trade. Previous work on China’s bird trade has relied predominantly on local market surveys, which are restricted to individual markets and capture only the species present at the time of survey. Judicial records cover the whole country and contain information on the identity of the traded birds, the purpose of the transaction, and the origin and destination of the trade. Our study focuses on three components: the taxonomic composition of the trade, the geographic structure of the trade network [28,29], and the motivations recorded for documented transactions, drawing on primary records from China Judgments Online [30]. We aim to draw attention to the domestic illegal bird trade and to encourage stronger regulatory oversight in key trading regions.

## 2. Materials and Methods

### 2.1. Data Collection and Categorization

We obtained judgment documents from China Judgments Online (https://wenshu.court.gov.cn/ (accessed on 10 January 2024)) by applying the platform’s filters in sequence. We first searched for the Chinese keyword ‘wildlife’ (野生动物), and the results were then filtered sequentially by case category, narrowing progressively from criminal cases to crimes of obstructing the administration of public order, to crimes of disrupting the protection of environmental resources, and finally to the crime of illegally acquiring, transporting, or selling rare and endangered wildlife and products thereof. The search was further restricted to judgments issued under first-instance criminal proceedings, and all judgments issued between 2014 and 2021 were downloaded and consolidated.

Following the methods used to collect and organize data on cross-border wildlife crimes in China [31], we extracted the following eight categories of information from these judgment documents. To reduce the uncertainty inherent in manual extraction, we established explicit extraction criteria and preserved the original wording of each document verbatim. All variables were coded from the verbatim text of the verdicts, and no inferences were drawn from information not stated in the documents. Records were restricted to mainland China (excluding Hong Kong, Macao and Taiwan), and smuggling cases were excluded:

(1) the date of the offence;
(2) the species traded;
(3) the consumer purpose of trade;
(4) the method of transaction;
(5) the transaction price of the traded birds;
(6) the officially assessed value of the traded birds;
(7) the number and status (live or dead) of the traded birds;
(8) the seller and the buyer of the traded birds.

The number of verdicts was defined as the number of judgment documents retrieved directly from China Judgments Online, and each trade event extracted from those verdicts was defined as one record. Verdicts were screened by offence date, so that only those involving offences committed between 2014 and 2021 were retained. The final dataset comprised 3416 records from 1427 verdicts.

We quantified the live individuals involved in illegal bird trade across different taxonomic categories based on records. In some records, only a combined total of live individuals was reported, without counts by order, because the original documents were ambiguous. We re-examined these verdicts and identified only two exceptions. One verdict recorded a total of 3696 live individuals of Anseriformes across 2015 and 2016, and we therefore used the annual mean for each of the two years. In another verdict, Psittaciformes and Columbiformes together accounted for 1941 live individuals, and we likewise averaged this total. These two verdicts represent 0.14% (2 of 1427) of the analyzed verdicts, and excluding them did not alter the ranking of orders by the number of live individuals.

Given the nature of the original verdicts, a single trade event may involve several traders linked in a chain of sellers and buyers, and may therefore appear in more than one verdict, so that the same birds could be counted more than once. At the same time, each of these verdicts documents a transaction that did in fact occur, so omitting them entirely would discard genuine records of trade. This is one of the reasons why we use the number of verdicts as the primary quantitative measure, rather than the number of individuals. For each case number, we extracted only the illegal trade case it addressed. Related cases mentioned under the same case number were not considered, so as to exclude potentially duplicated trade events.

### 2.2. Building the Network of China’s Illegal Bird Trade

We defined 261 Chinese prefectures, representing 233 prefecture-level cities, 22 autonomous prefectures, 1 prefecture, 1 league and 4 municipalities.

We generated chord diagrams to visualize inter-province trade flows of birds using the package circlize in R (4.4.2) [32]. To identify the key provinces and prefectures, we conducted social network analysis (SNA) and calculated degree centrality in Gephi (0.10.1 202301172018) [33]. SNA identifies influential nodes within an IWT network through centrality indices, and provinces or Chinese prefectures with high degree centrality occupy structurally important positions in the movement and distribution of illegal bird trade [34].

In social network analysis, degree centrality refers to the number of edges connected to a node. In our study, we distinguished in-degree (the number of edges entering a node) from out-degree (the number of edges leaving a node).

(1) $$d_{i}=\sum_{j}g_{\mathrm{ij}}$$

(2) $${\mathrm{wd}}_{i}=\sum_{j}w_{\mathrm{ij}}g_{\mathrm{ij}}$$

where d_i_ is the degree centrality of a node; g_ij_ is the number of connections from node i to node j; w_ij_ is the weight of connection from node i to node j; and wd_i_ is the weighted degree centrality of a node [35].

Each trading province or Chinese prefecture was represented as a node, and an edge was established between any two nodes between which a trade event was recorded. All networks were directed, with the origin of the traded birds as the source and the destination as the sink. Only trade routes with an unambiguous origin and destination were included. For each connection, the number of associated verdicts was used as the edge weight, and the weighted degree centrality of a node was obtained by summing the weights of all connections in which that province or Chinese prefecture was involved. Multiple trade events between the same origin–destination pair were aggregated into a single edge, whose weight was the cumulative number of verdicts recorded for that pair. Trade within the same administrative unit, where the origin and destination coincide and the edge becomes a self-loop, was weighted in the same way.

Betweenness centrality quantifies the extent to which a node lies on the shortest paths connecting all pairs of other nodes within a network. Formally, the higher the proportion of shortest paths between all node pairs in a network that pass through a given node, the higher its betweenness centrality. In this study, high betweenness centrality is interpreted only as structural importance within the observed network. However, a high betweenness value does not imply that traded birds physically passed through that province or Chinese prefecture.

(3) $${\mathrm{nb}}_{k}=\sum_{i}\sum_{j}\frac{g_{\mathrm{ikj}}}{g_{\mathrm{ij}}},i\neq j\neq k$$

where i, j, and k are the nodes of network; nb_k_ is the betweenness centrality of node k; and g_ikj_ is the number of shortest paths passing through node k.

We then calculated the geographic distances between all pairs of nodes involved in the illegal bird trade from their longitude–latitude coordinates using the geosphere package in R. These distances were grouped into <500 km (short), 500–1500 km (medium), and >1500 km (long) categories, and the number of verdicts in each category was counted to determine whether China’s illegal bird trade from 2014 to 2021 was dominated by short- or long-distance trade.

### 2.3. Distinguishing Consumer Demand of China’s Illegal Bird Trade

To analyze consumer motivations of illegal bird trade, we classified the trade purposes (the consumer purpose of trade). The initial collation of the above trade purposes revealed 21 categories: gift, trade, food, farm, pet, breeding, medicine, collection, domestication, exhibition, personal use, performance, release, research, report, save, crop protection, taxidermy, hunting, breed improvement and falconry.

Following Thomas-Walters et al. [27], we assigned 17 of the 21 trade purposes to four consumer demand categories (experiential, social, functional, and financial demand) (Table 1). Because each record captures a single seller-to-buyer transaction, we treat the buyer in each transaction as the consumer; any subsequent resale by that buyer is not recorded.

## 3. Results

### 3.1. The Scale of Illegal Bird Trade in China

#### 3.1.1. Species and Quantities Involved in China’s Illegal Bird Trade

**Figure 1:**
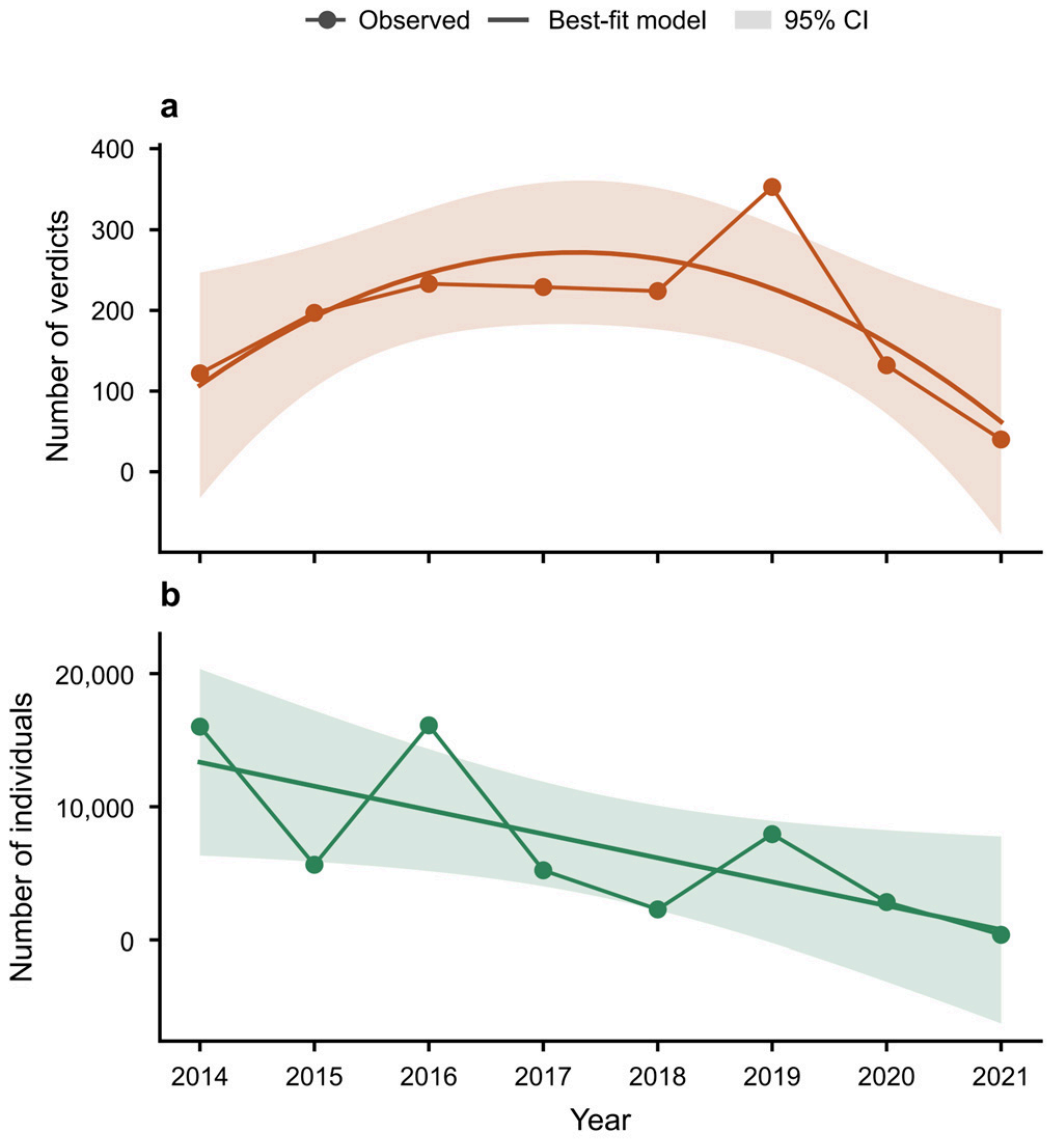
Temporal trends in China’s illegal bird trade from 2014 to 2021: (**a**) the number of verdicts; (**b**) the number of live individuals.

The number of illegal bird trade verdicts exhibited a distinct inverted U-shaped trajectory from 2014 to 2021 (Figure 1a). A quadratic polynomial regression indicated that verdict numbers rose from 122 in 2014 to a peak of 353 in 2019, before declining sharply to 40 in 2021. The quadratic model explained 66.2% of the variance in verdict numbers (R^2^ = 0.662, adjusted R^2^ = 0.526), and the quadratic term was statistically significant (*p* = 0.028). The total number of individual birds involved showed a significant overall downward trend over the same period (Figure 1b). Individual counts fluctuated from 16,013 in 2014 to 16,111 in 2016, fell markedly during 2017–2018 to 2286, rebounded briefly to 7939 in 2019, and then declined continuously to a minimum of 378 in 2021. Linear regression revealed a significant negative relationship between individual count and year (R^2^ = 0.535, *p* = 0.039), with year explaining approximately 53.5% of the variance. These analyses were based on only eight annual data points, a limitation that reduces statistical power.

**Figure 2:**
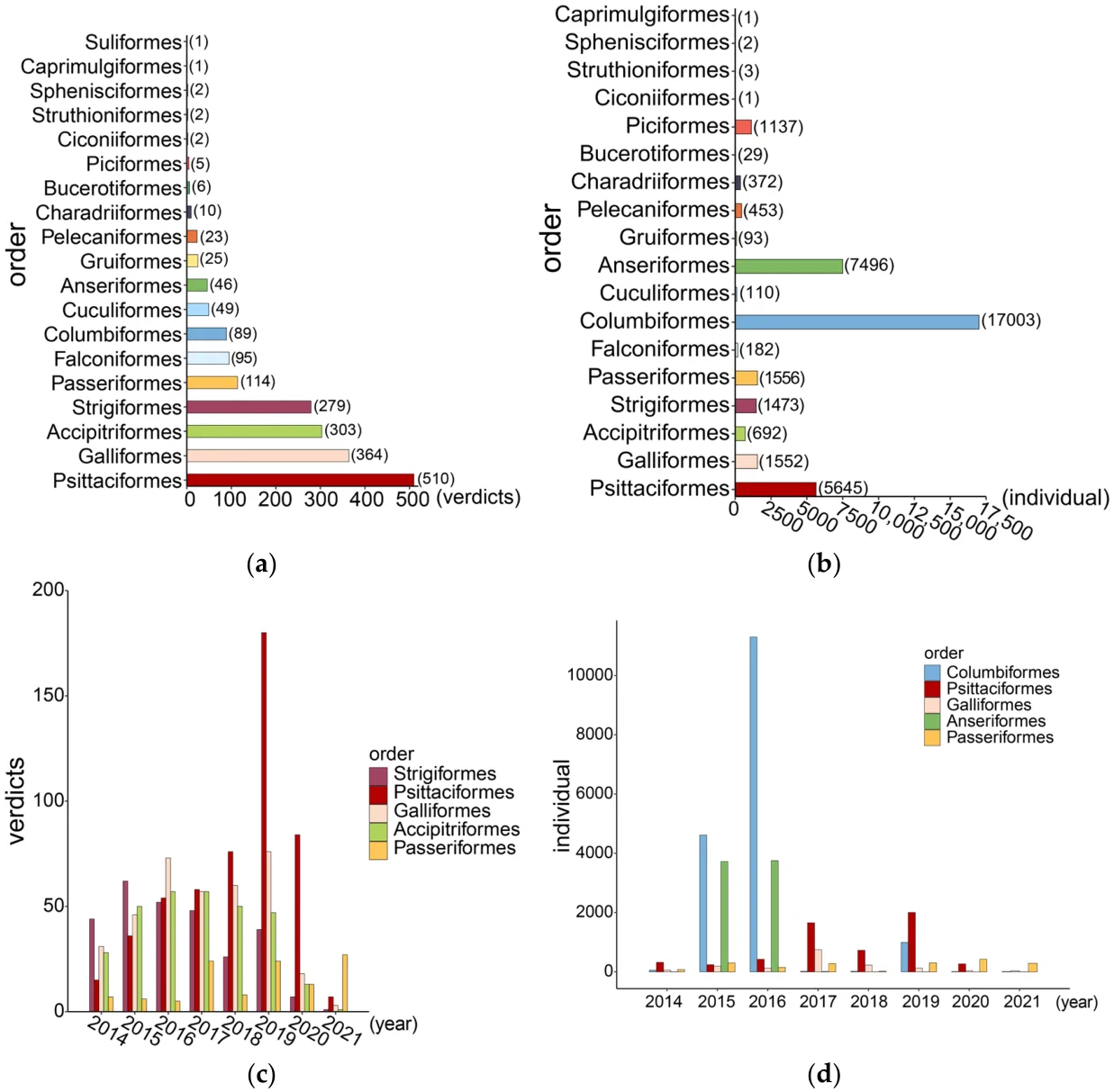
Distribution of verdict numbers and live individual counts by order in China’s illegal bird trade, 2014–2021: (**a**) number of verdicts involving the 19 bird orders; (**b**) number of live individuals involving the 19 bird orders; (**c**) annual verdict counts for the top five orders by number of verdicts, 2014–2021; (**d**) annual live individual counts for the top five orders by number of individuals, 2014–2021.

Overall, the illegal bird trade involved 48 families across 19 orders. Psittaciformes appeared in the greatest number of verdicts, followed by Galliformes, Accipitriformes, and Strigiformes. Passeriformes, Falconiformes, and Columbiformes showed comparable numbers of verdicts. Orders such as Suliformes, Caprimulgiformes, Sphenisciformes, Struthioniformes, and Ciconiiformes were rarely represented (Figure 2a).

Because verdict counts were low for most orders, we report annual counts separately for the five most frequently recorded orders (Figure 2c). The number of Strigiformes verdicts declined gradually from 2014 to 2021. The proportions of Galliformes and Accipitriformes verdicts remained relatively stable over this period, but began to decline from 2020. Verdicts involving Passeriformes increased year by year after 2019. Psittaciformes remained the most frequently traded group throughout, peaking in 2019.

Live individuals were recorded for 18 of the 19 orders, with Suliformes the only exception. Columbiformes accounted for the highest number of live individuals (17,003), followed by Anseriformes (7496) and Psittaciformes (5645), whereas Passeriformes and Galliformes showed comparable numbers (Figure 2b).

Columbiformes and Anseriformes were primarily documented in verdicts from 2015 and 2016, after which their involvement dropped sharply. Live Psittaciformes were recorded mainly in verdicts from 2017 to 2019, and most live Galliformes were recorded in 2017 (Figure 2d). Psittaciformes thus showed a pattern of many verdicts but few individuals, whereas Columbiformes showed the opposite pattern of few verdicts but many individuals, indicating that trade in Columbiformes was dominated by large, concentrated batches.

Based on the verdicts, 266 bird species were involved in China’s illegal bird trade from 2014 to 2021. Of these, 37 are assessed as threatened (CR, EN and VU) and 47 as Near Threatened (NT) on the Red List of China’s Vertebrates, 98 are listed as Least Concern (LC), and the remaining 84 are not assessed in that list. In the List of National Key Protected Wild Animals, 24 species are Class I and 83 are Class II protected. The most frequently recorded species were *Pyrrhura molinae* (127 verdicts), followed by *Myiopsitta monachus* (121), *Psittacus erithacus* (115), and *Lophura nycthemera* (112) (Table 2).

#### 3.1.2. Consumer Demand of China’s Illegal Bird Trade

Across the 2014–2021 judgments, financial demand involved 15 orders, functional demand involved 11 orders, experiential demand encompassed 10 orders, and social demand involved the 5 orders (Table S1).

**Figure 3:**
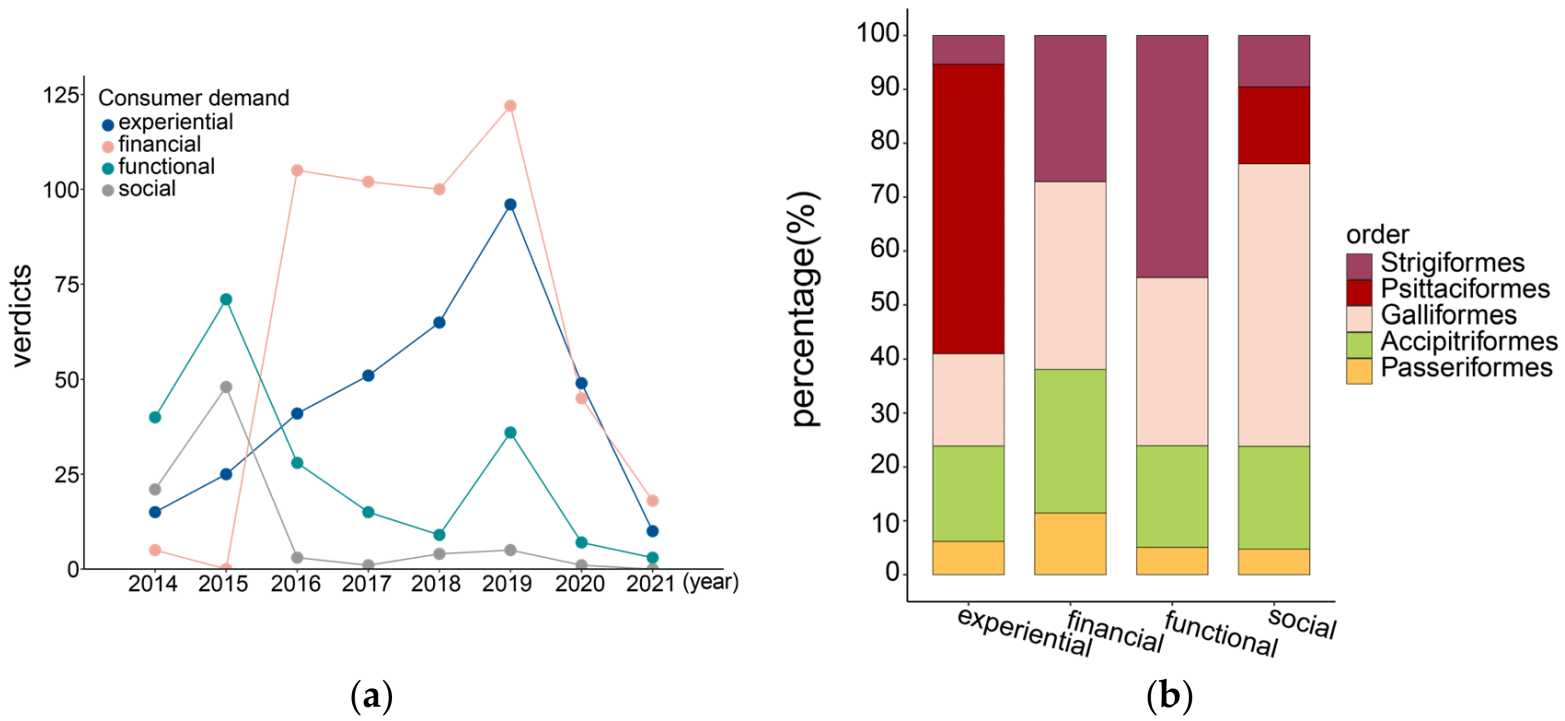
Consumer demand of illegal bird trade in China: (**a**) temporal trends in the four consumer demand categories; (**b**) percentage of verdicts for the five key orders within each consumer demand category. Percentages use, as the denominator, the total number of verdicts for these five orders in that category.

Verdict numbers for the four consumer demands showed distinct fluctuation patterns from 2014 to 2021 (Figure 3a; Table S2). Verdicts driven by financial demand represented the smallest proportion in 2014 and 2015 but increased substantially from 2016 onward and predominated in subsequent years; both experiential and financial demand peaked in 2019. Verdict numbers for functional and social demand showed similar patterns, both peaking in 2015.

When the five key orders were analyzed separately, Psittaciformes accounted for the largest proportion of experiential demand verdicts (53.65%). Strigiformes accounted for the largest proportion of functional demand verdicts (44.93%), and Galliformes recorded the highest number of verdicts in both financial and social demand (Figure 3b).

### 3.2. The Network of Illegal Bird Trade in China

#### 3.2.1. Provincial-Level Network

**Figure 4:**
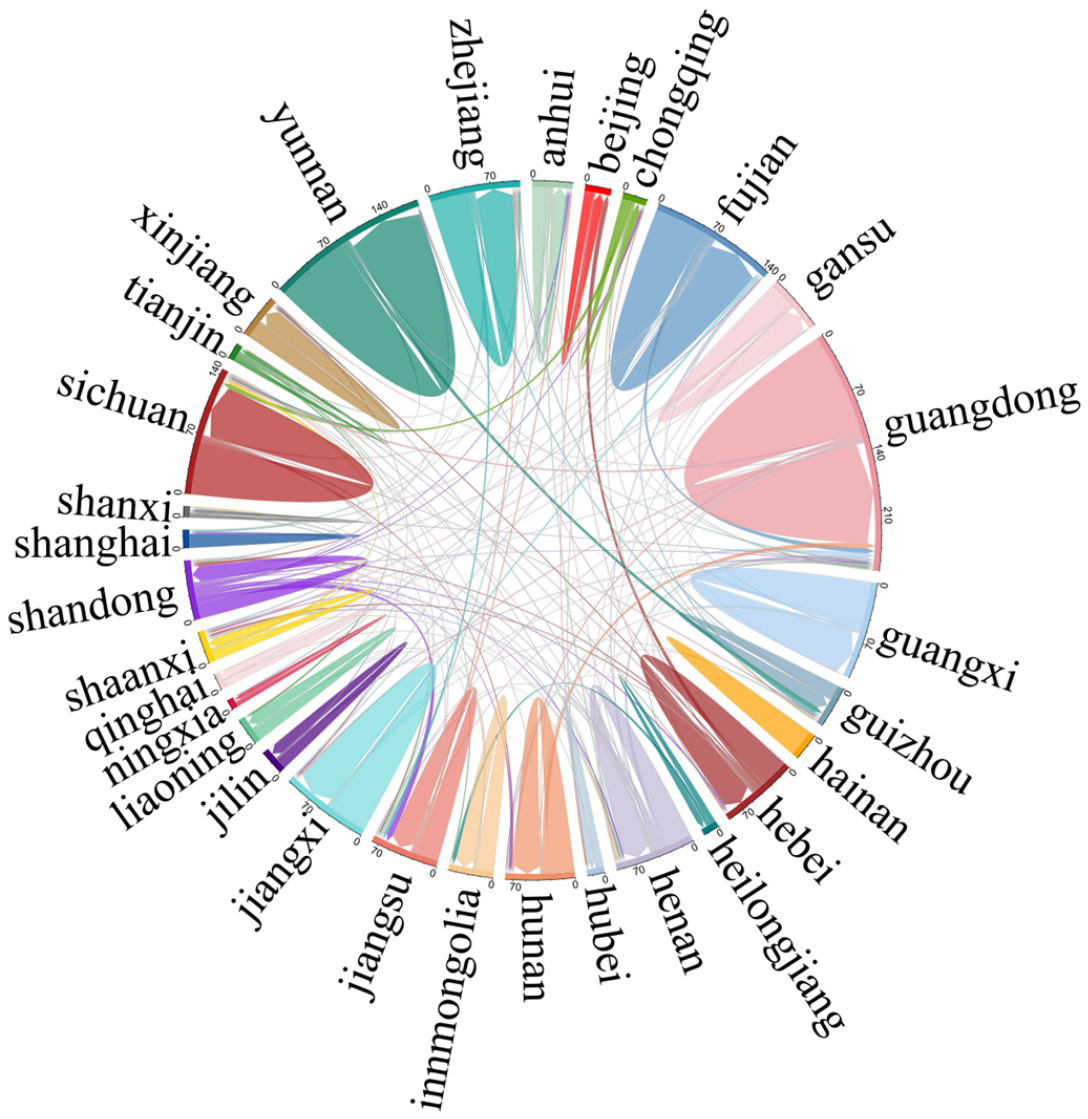
The network of China’s illegal bird trade among 30 provinces based on numbers of verdicts from 2014 to 2021: the chord diagram shows directional trade flows between provinces. Each outer sector represents a province, and its width is proportional to the total number of verdicts involving that province. Chords represent trade between provinces, with chord thickness proportional to the number of verdicts documenting that province pair; arrow direction indicates the direction of flow from origin to destination.

Illegal bird trade was concentrated in eastern, southwestern and southeastern China, and most transactions took place within a single province (Figure 4). Only a limited number of provinces engaged in frequent long-distance trade, with dense trade routes observed between Beijing in the north, Shanghai in the east, and Guangdong in the southeast (Figure S2).

The provinces with the highest numbers of verdicts were Guangdong, Yunnan, Sichuan, Fujian, and Guangxi, all located in southern and southwestern China (Table 3, Figure S3). To identify the primary buyers and sellers in the illegal bird trade network, we further distinguished weighted in-degree from weighted out-degree centrality (Table 3). Guangdong showed the highest weighted in-degree centrality, acting as the main destination market, followed by Yunnan, Sichuan and Fujian. Guangdong also showed the highest weighted out-degree centrality, acting as the primary source, followed by Yunnan, Fujian and Sichuan.

#### 3.2.2. Chinese Prefecture-Level Network

**Figure 5:**
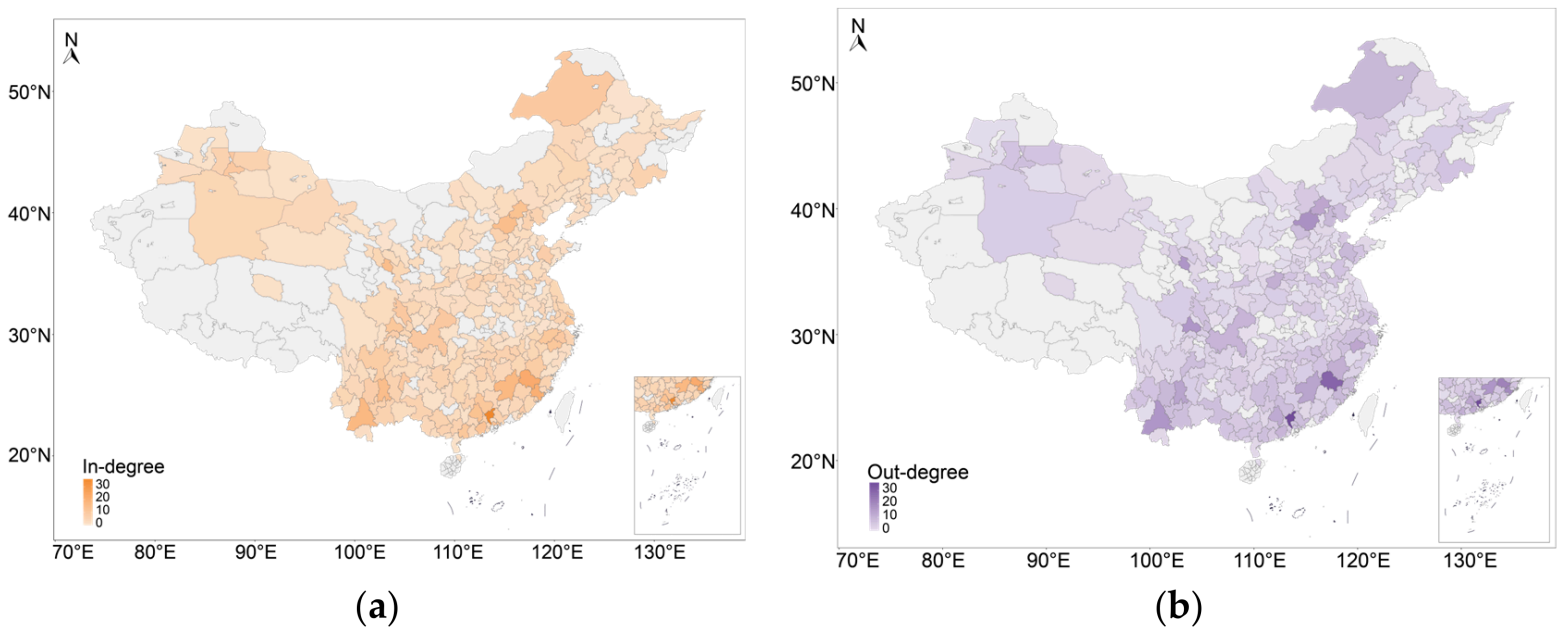
The degree centrality of Chinese prefectures: (**a**) weighted in-degree, representing the total number of illegal bird trade verdicts recorded for each city as a destination. (**b**) weighted out-degree, representing the total number of illegal bird trade verdicts recorded for each city as an origin. In both maps, color intensity ranges from pale to dark, and gray areas indicate cities with no recorded trade data.

At the Chinese prefecture level (Figure 5), Guangzhou exhibited the highest weighted degree, weighted in-degree and weighted out-degree. Almost all key Chinese prefectures were located in southeastern China (Table 4). Of the 1001 verdicts for which both the origin and the destination could be located precisely, 90.81% involved distances of less than 500 km, indicating that illegal bird trade in China between 2014 and 2021 was dominated by short-distance trade (Table S3; Figure S1).

#### 3.2.3. The Network of Five Key Orders

**Figure 6:**
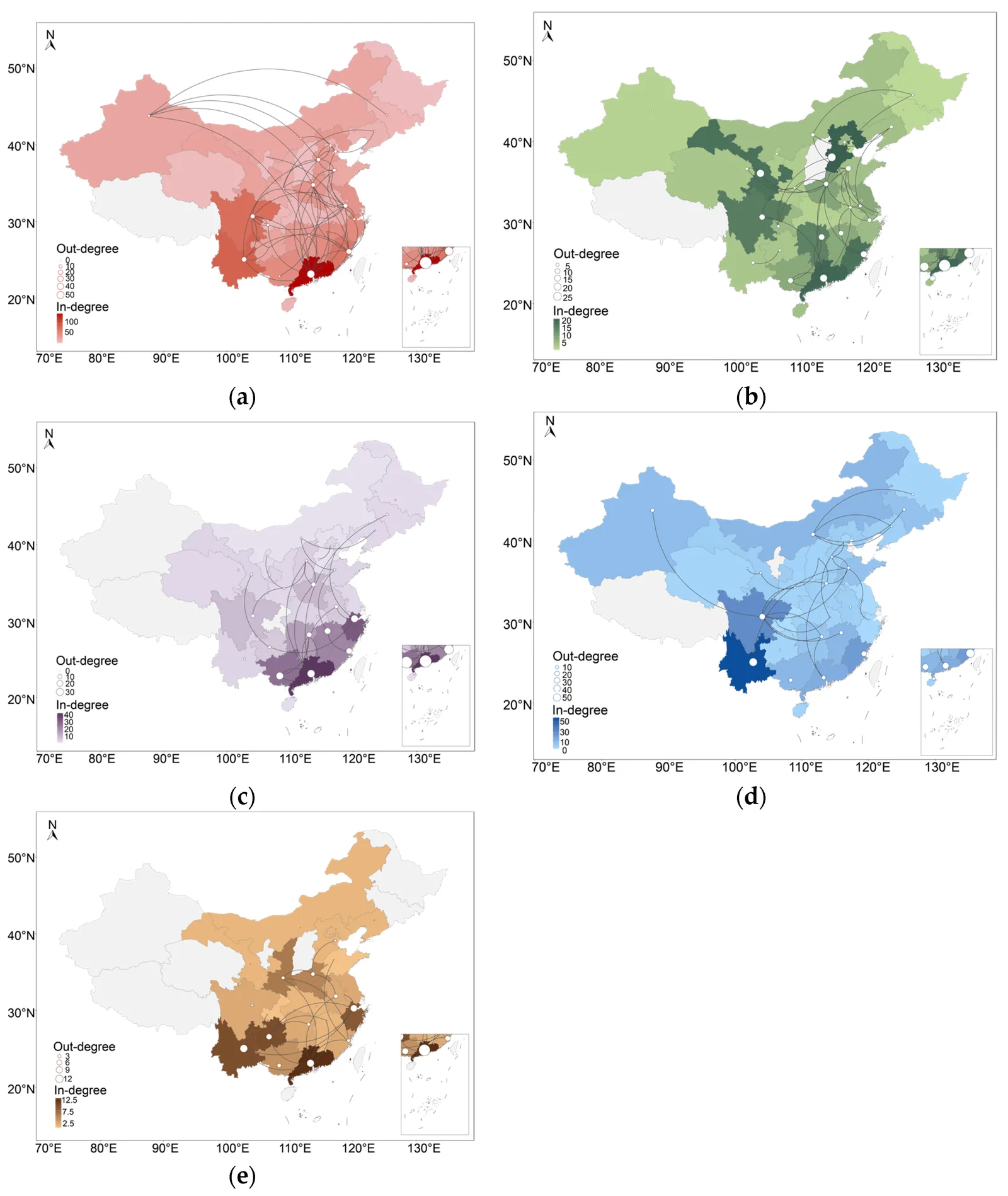
The weighted in-degree and out-degree centrality of provinces for five key orders: (**a**) Psittaciformes; (**b**) Galliformes; (**c**) Strigiformes; (**d**) Accipitriformes; (**e**) Passeriformes. Province color indicates weighted in-degree and circle size indicates weighted out-degree, both of which are weighted by the number of verdicts. Gray denotes provinces with no illegal bird trade verdicts.

We analyzed the provincial-level trade networks of the five orders with the highest numbers of verdicts (Psittaciformes, Galliformes, Strigiformes, Accipitriformes, and Passeriformes) (Figure 6).

The Psittaciformes network spanned 27 provinces. Guangdong recorded the highest weighted in-degree and weighted out-degree centrality, followed by Jiangsu, Sichuan, and Yunnan provinces. Fujian and Yunnan also showed relatively high weighted out-degree centrality.

Galliformes were traded illegally across 27 provinces, with no verdicts recorded in Tianjin, Shanghai, or the Ningxia Hui Autonomous Region. Yunnan showed the highest weighted in-degree and weighted out-degree centrality, with Sichuan and Fujian as the next most important source and destination nodes.

Illegal trade in Strigiformes occurred across 27 provinces. Guangdong recorded the highest weighted in-degree and weighted out-degree centrality, confirming its role as the most critical trade location for this order. Zhejiang and the Guangxi Zhuang Autonomous Region also showed substantial values in both centrality measures.

Accipitriformes showed the widest distribution across 29 provinces with only Shanxi excluded. Hebei exhibited higher weighted in-degree and weighted out-degree centrality than Guangdong, making it the predominant location for Accipitriformes trade.

Passeriformes were illegally traded in 22 provinces. Guangdong showed the highest weighted in-degree centrality, followed by Guizhou and Yunnan. Yunnan recorded the highest weighted out-degree centrality, with Guangdong and Zhejiang ranking second and third, respectively.

## 4. Discussion

Verdict counts should be interpreted as the number of documented and prosecuted cases rather than as a direct measure of the volume of illegal trade. They may be influenced by enforcement intensity, prosecution policy, and judicial practice. Individual counts are more directly informative about the number of birds involved but are subject to uncertainty from incomplete or ambiguous reporting. We therefore report both metrics.

The number of verdicts decreased sharply in 2020 and 2021. Field surveys of 12 bird markets across China also showed a notable decline in the proportion of wild birds [15]. This decline is potentially associated with the COVID-19 outbreak in 2020. In February 2020, the Standing Committee of the National People’s Congress issued a decision banning the farming, trade, and consumption of terrestrial wildlife for food [36], aiming to end indiscriminate wildlife consumption and to safeguard public health. This strict prohibition may have driven many wildlife breeders and traders out of business in China. Consistent with this, 89.5% of wildlife consumers reported reducing their wildlife consumption, and 70.5% of the public expressed support for banning all wildlife trade [37]. Whether the low number of verdicts recorded since 2021 will persist, however, remains uncertain and requires verification against subsequent annual data.

In the analyzed judicial records (2014–2021), illegal bird trade was prevalent across all provinces in China except the Tibet Autonomous Region [38]. With improving transportation infrastructure and the increasing accessibility of online platforms, the illegal bird trade in China may have evolved into a complex and highly interconnected criminal network.

Our study used degree centrality to identify provinces and Chinese prefectures that most urgently require intervention in illegal bird trade. This approach provides anchor points for understanding trade dynamics and dismantling trafficking networks, and it facilitates the development of targeted intervention strategies [39]. Within the network constructed here, Guangdong Province recorded the highest number of verdicts involving illegal bird trade and maintained trade connections with the most provinces, underscoring its role in facilitating transactions among individual traders and markets across China. This phenomenon may be attributed to Guangdong’s coastal location, its open, developed economic structure, and the historical prevalence of consuming wildlife meat as a cultural practice [40]. Because market prices for wildlife in Guangdong are high, a substantial share of the illegal wildlife from Guangxi is funneled into Guangdong for sale [41]. Targeting interventions in Guangdong, for example through enhanced market supervision and law enforcement, could therefore disrupt networks and curb poaching [42]. Southwestern China, meanwhile, has a high prevalence of wildlife trade and is recognized as a biodiversity hotspot [41]. Our findings corroborate this pattern, with Yunnan recording the second-highest number of verdicts after Guangdong. The prominence of Yunnan reflects two key factors: its exceptionally rich biodiversity [43] and its special geographic position [44], which facilitates cross-border trade.

In the analyzed network, Guangzhou recorded the highest number of illegal bird trade verdicts, making it a key node both within Guangdong and across China. Numerous live poultry markets operate in southern China, and despite awareness of the disease risks associated with purchasing live poultry, consumers in this region continue to prefer live birds [45]. The closure of markets during epidemics can even push trade into illegal channels, further complicating market regulation [46].

In the analyzed records, illegal bird trade in China occurred primarily locally or over short distances. This pattern may be attributed to the fact that illegal trade frequently takes place among acquaintances who have established stable trading relationships, while long-distance transportation entails higher costs and carries greater risks of detection.

In some impoverished regions, the sale of wildlife is a critical source of income [47]. In our data, the high proportion of financial demand may partly reflect the presence of intermediaries, because a purchaser who buys in order to resell is seeking financial gain in that transaction. Since each record covers a single transaction between one buyer and one seller, we cannot determine whether that buyer was the final consumer. Consumer demand is therefore defined at the level of the documented transaction rather than the end user. This definition remains valid because the purpose recorded in each verdict was stated by the party who acquired the wildlife and was not inferred by us. The demand expressed in that transaction is genuine market demand even if the animals were later resold. Psittaciformes are among the most popular birds kept as pets and traded commercially, representing one of the largest traded live bird populations in the world [48]. Their vibrant plumage, diverse sizes, and capacity for vocal mimicry [49] make them particularly appealing to consumers. In our study, Psittaciformes were not only the most frequently traded birds but also the group most often traded to meet experiential demand.

Consumer demand is a critical yet often overlooked dimension in IWT markets [26]. There is increasing awareness that wildlife poaching and trafficking represent a multifaceted problem that extends beyond mere law enforcement. Intensive regulation and law enforcement have proven inadequate to address the complexities of modern trade dynamics and fail to counteract the market forces driving IWT [50]. Therefore, it is imperative to shift attention to the consumer. In recent years, research has emphasized the need to focus on consumer demand and to develop behavioral interventions that reduce threats to wildlife populations [26,51]. Wildlife consumption begins with demand, which drives subsequent decisions and behavior. Understanding the underlying motivations can provide a theoretical foundation for changing consumer behavior toward wildlife [52,53,54] and ultimately reducing demand for wildlife.

The factors that trigger wildlife consumption are complex and may be related to culture, education level and conservation awareness. The precipitous decline of wildlife populations has created a perverse economic dynamic in which scarcity inflates value, turning wildlife consumption into luxury consumption. Because species perceived as rarer command higher market prices, conservation classifications and protective legislation may inadvertently accelerate their demise by increasing their value [55,56]. Changing public consumption behavior toward wildlife therefore requires integrated approaches combining education, social influence, regulation, and nudging. This complex challenge necessitates a multidisciplinary perspective, multi-stakeholder collaboration, and multi-scale approaches to develop effective solutions [53].

Several limitations should be acknowledged. First, our data are drawn from judicial records and therefore represent only those verdicts that were detected, prosecuted and posted online. They capture a fraction of illegal bird trade and should not be read as a direct measure of its true scale. Second, the number of available verdicts may vary geographically and temporally with enforcement intensity, prosecution policy and judicial practice, as well as with any variation in database coverage. Although judgments are posted under uniform national rules, we cannot exclude such biases. Third, because the assignment of trade purposes to consumer demand categories was judgmental rather than empirically derived, the consumer demand results provide only a preliminary indication of the consumer-side motivations documented in the verdicts and should not be taken as an accurate representation of the actual motivations of consumers in China’s illegal bird trade.

## 5. Conclusions

Based on the analyzed judicial records from 2014 to 2021, this study presents a systematic analysis of the illegal bird trade in China.

(1) The trade spanned 266 species, 19 orders and 48 families. Psittaciformes accounted for the largest number of verdicts, while Columbiformes, though seldom prosecuted, accounted for by far the largest number of live birds. Of these species, 37 are threatened and 107 are nationally protected (24 Class I and 83 Class II).
(2) Illegal bird trade was documented across 261 Chinese prefectures in 30 provinces. Guangdong acted simultaneously as the largest supplier and the largest market, and among prefectures Guangzhou was the most central node. Among the 1001 verdicts for which both the origin and destination were identified, 90.81% involved trade distances of less than 500 km, indicating that illegal bird trade was dominated by short-distance trade.
(3) Psittaciformes were the most frequently traded order and were traded mainly to meet experiential demand. Galliformes emerged as the second most traded group, serving primarily to meet financial demand.

The recurrently traded taxa and highly connected geographic nodes identified here can help guide monitoring and enforcement priorities, provided that the limitations inherent in judicial-record data are taken into account.

## Figures and Tables

**Table 1:** Classification of purposes and consumer demand in China’s illegal bird trade, 2014–2021.

Trade Purpose	Consumer Demand
trade	Financial (desire for financial gain or immediate profit)
performance	
taxidermy	
breeding	
farm	
breed improvement	
food	Functional (need to fulfill an everyday purpose or function)
medicine	
research	
crop protection	
hunting	
gift	Social (desire to enhance social status or gain social recognition)
collection	
exhibition	Experiential (fulfill hedonistic pleasure, provide novelty, or satisfy curiosity)
domestication	
pet	
falconry	
personal use	—
release	—
report	—
save	—

**Table 2:** Top 10 species by number of verdicts in China’s illegal bird trade, 2014–2021.

Family	Genus	Species	Number of Verdicts (n)
Psittacidae	Pyrrhura	*Pyrrhura molinae*	127
Psittacidae	Myiopsitta	*Myiopsitta monachus*	121
Psittacidae	Psittacus	*Psittacus erithacus*	115
Phasianidae	Lophura	*Lophura nycthemera*	112
Accipitridae	Accipiter	*Accipiter gentilis*	95
Psittacidae	Aratinga	*Aratinga solstitialis*	90
Phasianidae	Chrysolophus	*Chrysolophus amherstiae*	71
Strigidae	Otus	*Otus lettia*	65
Phasianidae	Chrysolophus	*Chrysolophus pictus*	63
Leiothrichidae	Garrulax	*Garrulax canorus*	54

**Table 3:** Key provinces identified by the network indicators.

Province	Weighted Degree	Province	Weighted In-Degree	Province	Weighted Out-Degree	Province	Betweenness Centrality
Guangdong	278	Guangdong	147	Guangdong	131	Guangdong	0.3902
Yunnan	185	Yunnan	89	Yunnan	96	Henan	0.2151
Sichuan	147	Sichuan	78	Fujian	75	Hebei	0.2026
Fujian	145	Fujian	70	Sichuan	69	Shandong	0.1169
Guangxi	111	Zhejiang	52	Guangxi	60	Tianjin	0.0991
Zhejiang	104	Guangxi	51	Zhejiang	52	Jiangsu	0.0842
Jiangxi	101	Jiangxi	51	Jiangxi	50	Shaanxi	0.0734
Henan	92	Jiangsu	45	Henan	50	Sichuan	0.0719
Hebei	89	Henan	42	Hebei	50	Fujian	0.0655
Hunan	79	Hunan	40	Shandong	42	Mongolia	0.0531

**Table 4:** Key Chinese prefectures identified by the network indicators.

Prefecture	Weighted Degree	Province	Weighted In-Degree	Province	Weighted Out-Degree	Province	Betweenness Centrality
Guangzhou	72	Guangzhou	36	Guangzhou	36	Chengdu	0.0346
Sanming	52	Sanming	22	Sanming	30	Shantou	0.0341
Pu’er	34	Quanzhou	20	Baoding	18	Baoding	0.033
Baoding	32	Ganzhou	17	Chengdu	17	Guangzhou	0.0325
Ganzhou	31	Pu’er	17	Pu’er	17	Yangjiang	0.0323
Linxia Hui Autonomous Prefecture	31	Kunming	15	Linxia Hui Autonomous Prefecture	17	Cangzhou	0.0285
Kunming	29	Baoding	14	Kunming	14	Huizhou	0.0276
Quanzhou	29	Linxia Hui Autonomous Prefecture	14	Ganzhou	14	Zhangzhou	0.0259
Chengdu	28	Shenzhen	13	Jinhua	13	Chaozhou	0.0257
Beijing	26	Beijing	13	Beijing	13	Liangshan Yi Autonomous Prefecture	0.0231

## Data Availability

The datasets presented in this article are not readily available because the data are part of an ongoing study or due to time limitations. Requests to access the datasets should be directed to the corresponding author upon reasonable request.

## Supplementary Materials

The following supporting information can be downloaded at https://www.mdpi.com/article/10.3390/biology15212015/s1, Figure S1: Histogram of the distance of China’s illegal bird trade; Figure S2: The network pattern of China’s illegal bird trade; Figure S3: The degree centrality of provinces: (a) the weighted in-degree; (b) the weighted out-degree; Table S1: The number of verdicts involving four consumer demands by orders; Table S2: The number of verdicts involving four consumer demands by years; Table S3: Percentage of verdicts in each trade-distance class (only verdicts with a geolocatable origin and destination were included).
