# Supplementary material for: Taxonomic Composition, Trade Networks, and Consumer Demand in China’s Illegal Bird Trade: Evidence from Court Verdicts (2014–2021)
Source: Biology (Basel). 2026 Nov 5;15(21):2015. doi: 10.3390/biology15212015 (PMC13762351; doi:10.3390/biology15212015)
Supplement: Supplementary file 1 [file biology-15-02015-s001.zip › biology-4582168-supplementary.pdf]

Species Composition, Trade Networks, and Consumer Demand in China's Illegal Bird Trade: Evidence from Verdicts (2014–2021)

Supplementary Files

- **Figure S1:** Histogram of the distance of China’s illegal bird trade
- **Figure S2:** The network pattern of China’s illegal bird trade
- **Figure S3:** The degree centrality of provinces: (a) the weighted in-degree; (b) the weighted out-degree.
- **Table S1:** The number of verdicts involving four consumer demands by orders
- **Table S2:** The number of verdicts involving four consumer demands by years
- **Table S3:** Percentage of verdicts in each trade-distance class (only verdicts with a geolocatable origin and destination were included)

Supplementary Figures

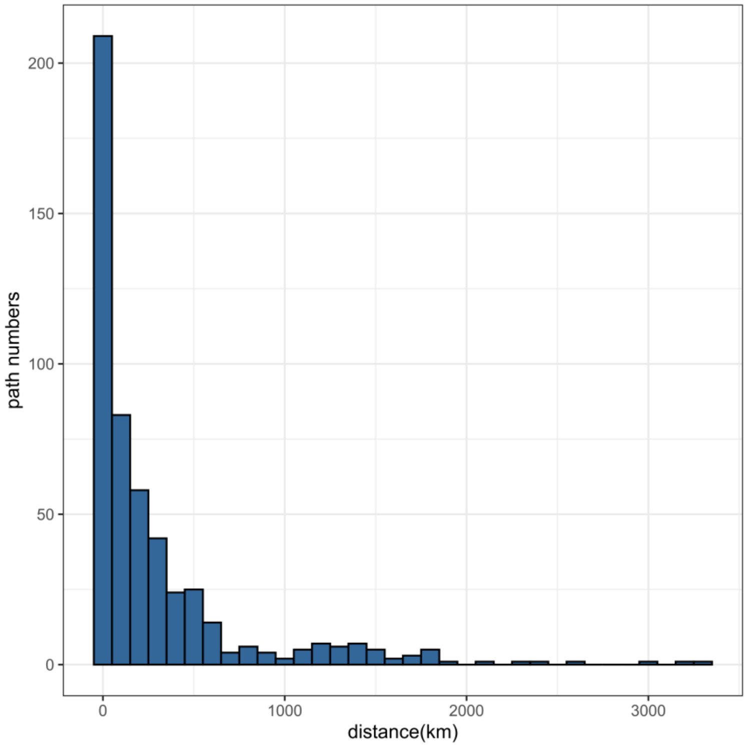

Figure S1 Histogram of the distance of China's illegal bird trade

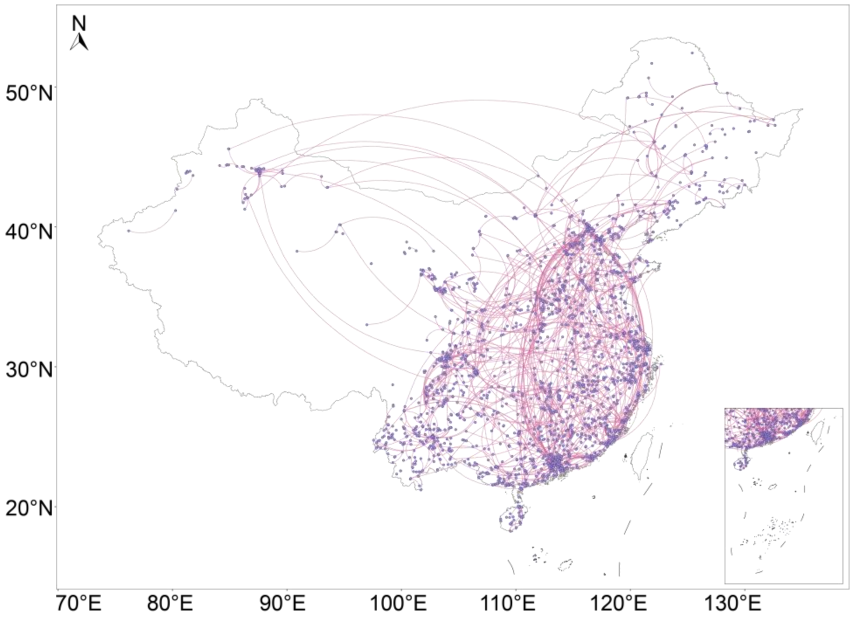

Figure S2 The network pattern of China's illegal bird trade

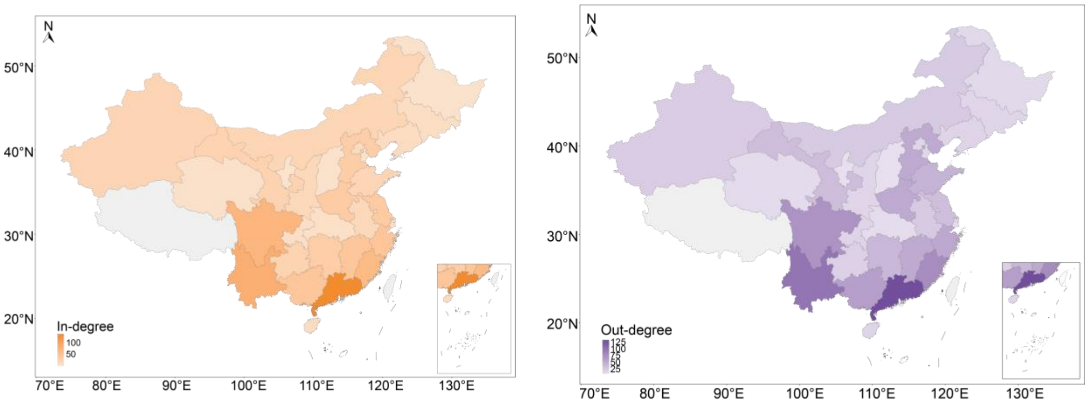

(**a**) (**b**)

Figure S3. The degree centrality of provinces: (a) the weighted in-degree; (b) the weighted out-degree.

Supplementary Tables

Table S1 The number of verdicts involving four consumer demands by orders

Consumer demand	Order	N court verdicts
Financial	Galliformes	155
	Strigiformes	121
	Falconiformes	33
	Accipitriformes	119
	Cuculiformes	23
	Passeriformes	51
	Anseriformes	23
	Gruiformes	17
	Columbiformes	28
	Pelecaniformes	10
	Charadriiformes	3
	Bucerotiformes	2
	Piciformes	2
	Ciconiiformes	2
	Struthioniformes	1
Functional	Galliformes	43
	Strigiformes	62
	Falconiformes	6
	Accipitriformes	26
	Cuculiformes	13
	Passeriformes	7
	Anseriformes	5
	Gruiformes	1
	Columbiformes	3
	Pelecaniformes	5
	Charadriiformes	1
Experiential	Galliformes	61
	Strigiformes	19
	Falconiformes	24
	Accipitriformes	63
	Cuculiformes	3
	Passeriformes	22
	Psittaciformes	191
	Gruiformes	1
	Columbiformes	6
	Struthioniformes	1
Social	Galliformes	11
	Strigiformes	2
	Accipitriformes	4
	Passeriformes	1
	Psittaciformes	3

Table S2 The number of verdicts involving four consumer demands by years

	**Functional**	**Financial**	**Social**	**Experiential**
**2014**	40	5	21	15
**2015**	71	0	48	25
**2016**	28	105	3	41
**2017**	15	102	1	51
**2018**	9	100	4	65
**2019**	36	122	5	96
**2020**	7	45	1	49
**2021**	3	18	0	10

Table S3 Percentage of verdicts in each trade-distance class (only verdicts with a geolocatable origin and destination were included)

Distance class (km)	Verdicts (n)	Percentage of verdicts
Short (＜500)	909	90.81%
Medium (500 - 1500)	71	7.09%
Long (＞1500)	21	2.10%
